# BJLD-CMI: a predictive circRNA-miRNA interactions model combining multi-angle feature information

**DOI:** 10.3389/fgene.2024.1399810

**Published:** 2024-05-10

**Authors:** Yi-Xin Zhao, Chang-Qing Yu, Li-Ping Li, Deng-Wu Wang, Hui-Fan Song, Yu Wei

**Affiliations:** ^1^ School of information Engineering, Xijing University, Xi’an, China; ^2^ College of Grassland and Environment Sciences, Xinjiang Agricultural University, Ürümqi, China

**Keywords:** circRNA-miRNA interaction, circRNA, miRNA, graph embedding, autoencoder

## Abstract

Increasing research findings suggest that circular RNA (circRNA) exerts a crucial function in the pathogenesis of complex human diseases by binding to miRNA. Identifying their potential interactions is of paramount importance for the diagnosis and treatment of diseases. However, long cycles, small scales, and time-consuming processes characterize previous biological wet experiments. Consequently, the use of an efficient computational model to forecast the interactions between circRNA and miRNA is gradually becoming mainstream. In this study, we present a new prediction model named BJLD-CMI. The model extracts circRNA sequence features and miRNA sequence features by applying Jaccard and Bert’s method and organically integrates them to obtain CMI attribute features, and then uses the graph embedding method Line to extract CMI behavioral features based on the known circRNA-miRNA correlation graph information. And then we predict the potential circRNA-miRNA interactions by fusing the multi-angle feature information such as attribute and behavior through Autoencoder in Autoencoder Networks. BJLD-CMI attained 94.95% and 90.69% of the area under the ROC curve on the CMI-9589 and CMI-9905 datasets. When compared with existing models, the results indicate that BJLD-CMI exhibits the best overall competence. During the case study experiment, we conducted a PubMed literature search to confirm that out of the top 10 predicted CMIs, seven pairs did indeed exist. These results suggest that BJLD-CMI is an effective method for predicting interactions between circRNAs and miRNAs. It provides a valuable candidate for biological wet experiments and can reduce the burden of researchers.

## 1 Introduction

Circular RNAs (circRNAs) are a new class of endogenous non-coding RNAs with covalently closed loops that are important components of gene transcription. In comparison to traditional linear RNA, circRNA, due to it is end-to-end covalent closure and the absence of a 5′ cap or 3′ poly(A) tail (Zheng et al., 2020), is less susceptible to degradation by exonucleases, rendering it is structure more stable. CircRNA was first discovered in virus-infected plant particles in 1976. However, due to it is low expression levels and sparse occurrence, it was initially considered a byproduct of gene transcriptional splicing errors or “splicing noise,” receiving limited attention from researchers at that time. Hsu and Coca-Prados (1979) used electronic microscopy in 1979 to provide indications of the existence of circRNA in the cytoplasm of eukaryotic cell lines. With the maturity of high-throughput sequencing technology as well as the continuous development of bioinformatics, researchers have discovered that circRNA is abundant in eukaryotes and performs a crucial role in various biological processes. In 2013, Memczak et al. (2013) demonstrated through sequence analysis that circRNA has important regulatory functions. In the same year, Hansen et al. (2013) discovered a highly expressed circular RNA that binds with miR-7 within human and mouse brain. Additionally, they identified a testis-specific circRNA acting as a miR-138 sponge. This led to the inference that the miRNA sponge effect formed by circRNA is a widespread phenomenon. Simultaneously, various biological functions of circRNA have been gradually understood by humans. For instance, circRNA can act as a scaffold for protein complex assembly, regulate gene expression, and modulate selective splicing RNA-protein interactions (Deng et al., 2019). As understanding of circRNA and miRNA deepens, an increasing amount of research evidence suggests the existence of connections between the two. CircRNAs participate in organic processes, and their dysregulation and mutations can affect disease progression. As an example, Gong et al. (2023) discovered that hsa_circ_0064644 could inhibit the proliferation and migration of osteosarcoma cells by acting as a miR-424-5p sponge to regulate eIF4B and YRDC. Wang et al. (2022a) demonstrated that hsa_circ_0005505 modulates KIF2A expression by acting as a miR-603 sponge, and silencing hsa_circ_0005505 will cause self-apoptosis of breast cancer cells, which cannot normally multiply, move and invade other tissues *in vitro*. Causing tumor growth in the body to slow down. Pan et al. (Pan and Binghua, 2023) confirmed that hsa_circ_0135761 positively regulates EFR3 by competitively binding to miR-654-3p. Reducing the gene level of hsa_circ_0135761 promotes apoptosis in NP carcinoma cells, as well as inhibits the increase and relocation of nasopharyngeal carcinoma cells. Therefore, circRNA may serve as a potential biomarker. Examining the potential correlation between circRNAs and miRNAs holds significant clinical guidance for biologists in diagnosing and treating diseases.

Before the popularity of computational models, identifying the relation of circRNAs to miRNAs typically relied on classical biological experimental methods. However, validating these experimental results often proved to be quite cumbersome. With the discovery of an increasing number of circular RNAs, the growing number of miRNAs to be validated has posed significant challenges to traditional biological experimental verification methods. As computational models gradually address the drawbacks of traditional biological experiments, including long experimental cycles, high costs, small-scale studies, and susceptibility to external interference, an increasing number of researchers have begun to predict the interrelationships between circRNAs and miRNAs with the help of computational modeling. This alleviates the burden of experimental validation and provides researchers with a broader perspective. In 2018, Li et al. (2018) explored data integration principles using a machine learning approach to analyze a variety of downstream tasks using a computer-based perspective. In 2019, the computational framework CMASG was introduced by Qian et al. (2021b), which utilizes singular value decomposition and graph variational autoencoder to extract linear and nonlinear features from circRNA and miRNA. Additionally, they integrated the framework to predict the interactions between them. In 2021, Lan et al. (2021) suggested a new method named NECNA for network-based embedding. This method utilizes GIP kernel similarity networks of circRNA and miRNA, along with their associated networks, to construct a heterogeneous network. Through neighborhood regularized matrix factorization and inner product, NECNA predicts the interaction between circRNA and miRNA.

As methods for extracting and fusing features continue to mature, a single feature cannot interpret all the information in an organism, so researchers have begun to combine multiple features to interpret information in organisms. In 2021, Qian et al. (2021a) proposed a model, MKSVM, which fuses multiple feature information extracted from protein sequences through a central kernel alignment-based multiple kernel learning (MKL-CKA) algorithm to predict DBP. In 2022, a computational model called WSCD was introduced by Guo et al. (2022). It utilizes graph embedding and word embedding to extract features and integrates convolutional neural networks (CNN) and deep neural networks (DNN) to deduce the potential interactions between circRNA and miRNA. In the same year, Qian et al. (2022a) proposed a prediction model for adverse drug reactions, which constructs two spatial RBMs to predict drug-side effect associations by fusing the similarity feature matrix of the drug chemical structure information and the similarity feature matrix of the association mapping based on the central kernel alignment (MKL-CKA) algorithm, as well as the adjacency matrix supplemented by Weighted K nearest known neighbors. Yu et al. (2022) introduced a model named SGCNCMI. This model employs a graph neural network with a contribution mechanism to aggregate multi-modal information from nodes for predicting CMIs. Qian et al. (2022b) proposed a model, MvKSRC, which combines multi-view features such as amino acid composition, evolutionary information and amino acid physicochemical information to further predict membrane protein types by Kernel Sparse Representation based Classification (KSRC). Wang et al. (2022b) designed a computational method, KGDCMI, based on the fusion of multiple sources of information to predict interactions between circRNA and miRNA. This method combines sequence and similarity to obtain attribute features, combined with the behavior features, the extracted feature vectors are sent to the deep neural network for prediction. In 2023, Li et al. (2023) introduced a multi-source information fusion model, DeepCMI. This model integrates various information, including sequence similarity matrices and Gaussian interaction kernel features, to construct multi-source features. Through linear embedding prediction of CMI by enhanced feature extraction through linear embedding.

**FIGURE 1:**
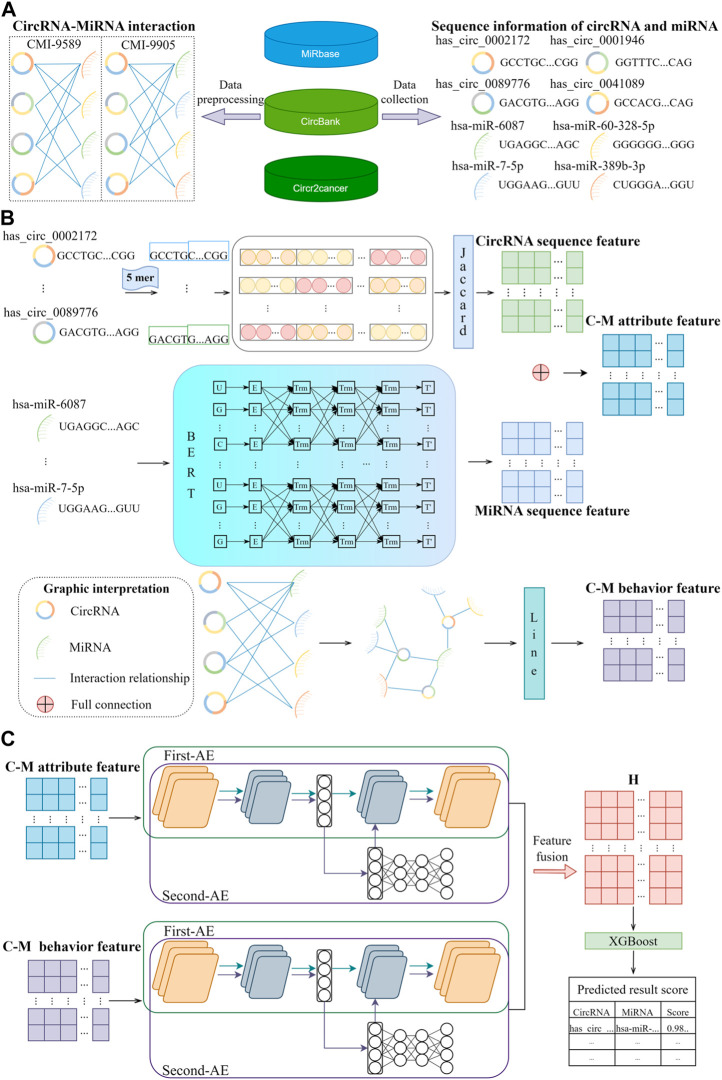
BJLD-CMI workflow diagram. **(A)** Data were collected and cleaned from MiRbase, CircBank, and CircR2cancer databases to obtain the CMI-9589 dataset and CMI-9905 dataset, respectively. **(B)** Jaccard, Bert, and Line were applied to extract the known attribute features and behavioral features of CMI. **(C)** Use Autoencoder in Autoencoder Networks to fuse the attribute features and behavioral features, and the fused features are predicted and analyzed by the XGBoost classifier for each CMI.

Although the existing models mentioned above have achieved relatively influential prediction results, they still inevitably have certain limitations in terms of efficiency and methodology, and many prediction models are built on statistical models and machine learning algorithms that lack an understanding of biological information. Consequently, the prediction results may be difficult to interpret or unreliable. To address the above issues, we proposed a novel computational model-based approach called BJLD-CMI for predicting circRNA-miRNA interactions in this study. Firstly, We utilized the Jaccard and Bert (Devlin et al., 2018) methods to extract features from the circRNA and miRNA sequences respectively and organically integrate them into attribute features of CMI. Secondly, we employed the graph embedding method Line (Tang et al., 2015) to extract behavioral features of CMI based on the graph information of circRNA-miRNA interactions. We then introduce the Autoencoder in Autoencoder Networks (Zhang et al., 2019) model to fuse the behavioral and attribute features of circRNA and miRNA, obtaining comprehensive features between them. Finally, an XGBoost (Chen and Guestrin, 2016) classifier is utilized to predict potential CMIs. We conducted a comprehensive evaluation of the model performance based on five-fold cross-validation (5-fold CV). In the validation on the CMI-9905 dataset, we achieved remarkable performance, with the AUC reaching 90.69%, accuracy as high as 88.36%, and precision reaching 85.31%. We also compared different classifiers and obtained favorable results. Additionally, we conducted a case study on BJLD-CMI, wherein we validated the top 10 predicted CMI pairs from the experiment through the latest literature in the PubMed database. It was found that 7 of these pairs have already been confirmed to have a relationship. Based on these experimental results, we conclude that the BJLD-CMI model plays a significant role in predicting the interaction between circRNA and miRNA, providing effective guidance for biological experiments to identify circRNA as relevant miRNA sponges. Figure 1 illustrates the workflow of BJLD-CMI.

## 2 Materials and methods

### 2.1 Dataset

For this study, to assess the model’s ability to predict CMI, we utilized two commonly used datasets, namely, the CMI-9589 dataset and the CMI-9905 dataset. The CMI-9589 dataset is sourced from the CircBank (Liu et al., 2019) database, which is a comprehensive human circRNA database containing detailed annotations for 140,790 human circRNAs from various sources. In addition to providing basic information about circRNA, CircBank also offers a set of interaction data between circRNA and miRNA for predicting and analyzing miRNA interactions. Based on the cleaning and summarizing the data, we acquired known 9,589 circRNA-miRNA pairs, among which 2,115 circRNAs and 821 miRNAs were involved. The CircR2Cancer (Lan et al., 2020) database is a manually curated database that associates circRNA with cancer. The CMI-9905 dataset, obtained by Wang et al. (2022b), comprises data on circRNA-miRNA interactions from the public database CircR2Cancer, including combined 318 circRNA-miRNA pairs among 238 circRNAs and 230 miRNAs. By integrating the data of the two databases, 9,905 good-quality CMI pairs were final acquired, comprising 2,346 circRNAs and 962 miRNAs. In this study, we primarily utilized the CMI-9905 dataset and regarded it as the positive sample, detailed information is available in Table 1. Subsequently, we randomly selected 9,905 unknown CMI pairs from the data pool of 2346×962−9905=2,246,947 as negative samples.

**TABLE 1:** CMI data set information used in BJLD.

Dataset	CircRNA	MiRNA	Interaction
CMI-9589 (CircBank)	2,115	821	9,585
CMI-9905 (CircBank, Circr2cancer)	2,346	962	9,905

### 2.2 Constructing attribute characteristics

In bioinformatics, researchers typically analyze the nucleotide sequences of RNA to extract features such as nucleotide composition, base pair frequency, sequence length, etc. These features help reveal the structure, function, and relationships with other biomolecules of RNA. Due to the substantial differences in the length of circRNA nucleotide sequences, with some long circRNAs containing thousands of nucleotides and short circRNAs containing only a few hundred nucleotides, we chose to use the Jaccard similarity coefficient to extract attribute features from circRNA sequences. This is because the Jaccard similarity coefficient can better reflect the similarity between sequences of different lengths. miRNAs usually have relatively short lengths, typically between 20 and 22 nucleotides. To extract attribute features from miRNA sequences, we employed the Bert model. Finally, we integrate the attribute features of circRNAs and miRNAs depending on the interaction relationships and finally obtain the CMI attribute features of known relationship pairs.

#### 2.2.1 Jaccard similarity coefficient

The Jaccard model is one of the fundamental models for similarity recognition, while the Jaccard similarity coefficient is an important metric used to measure the similarity between two sets. The factor takes values between 0 and 1, and the closer the value is to 1, the more similar the two sets are. The Jaccard similarity coefficient, widely employed in bioinformatics, serves to assess the dissimilarity or similarity between finite sets of samples (Wang et al., 2021). In order to extract the most effective sequence information, we use a moving window whose window size is 5 and whose stride size is 1 to split the sequence. This divides a circRNA of length L into sets of length L/5 . Then, we utilize the Jaccard similarity coefficient to gauge the representation of differences in circRNA $C_{a}$ and other circRNA sequences in the sample, represented as Formula 1:

$$J_{C_{a}}=\left|\frac{C_{a}\cap C_{e}}{C_{a}\cup C_{e}}\right|$$ (1)

Where $C_{e}$ represents the sequence set of 2,346 different circRNAs, with $e\in \left[1,2346\right]$ .

#### 2.2.2 Natural language processing model of bert

**FIGURE 2:**
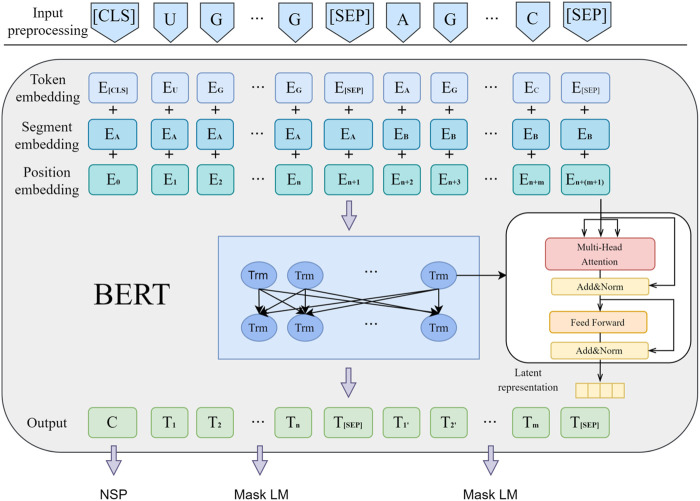
Basic flowchart of Bert pre-training.

Bert (Devlin et al., 2018) is an unsupervised pre-trained language model used for natural language processing tasks. It consists a bidirectional multi-layer Transformer encoder stack. Bert learns from two unsupervised pre-training tasks, namely, Masked Language Model (MLM) and Next Sentence Prediction (NSP) tasks. In the MLM task, model learning anticipates some tokens of the input sequence by shadowing them randomly. In the NSP task, model learns to judge whether two sentences were adjacent or not. As shown in Figure 2, Bert preprocesses the input information, represented as Formula 2 when the input is miRNA sequence information:

$${input}_{pre}=\left(\left[CLS\right],U,G,\ldots ,\left[SEP\right],A,G,\ldots ,C,\left[SEP\right]\right)$$ (2)

$\left[CLS\right]$ is a special token added at the beginning of each input sequence, and $\left[SEP\right]$ is a special token used as a separator. Bert’s input is the sum of Token embedding, Segment embedding, and Position embedding, represented as Formula 3:

$$\begin{array}{c} input={Embedding}_{Tok}\left({input}_{pre}\right)+{Embedding}_{Seg}\left({input}_{pre}\right)\\ +{Embedding}_{Pos}\left({input}_{pre}\right) \end{array}$$ (3)

At the encoding layer, Bert comprises a 12-layer Transformer encoding network, with each layer having a hidden size of 768, aimed at extracting latent features and establishing correlations between contexts. Compared to ELMo (Sarzynska-Wawer et al., 2021), Bert uses Transformer blocks as extractors, pre-trained through MLM (Masked Language Model) to enhance semantic feature extraction capabilities. In contrast to GPT (Radford et al., 2018), Bert switches from unidirectional to bidirectional encoding, leveraging all context information for each word and predicting and reconstructing the original data from corrupted input data through autoencoding. Compared to unidirectional encoders that can only utilize leading information for semantic information extraction, Bert exhibits stronger semantic information extraction capabilities.

In this study, we obtained a set of 9,905 relationship pairs between circRNA and miRNA, involving the IDs of 962 miRNAs. To study these miRNAs in depth, we introduced that Bert model. By querying miRbase (Griffiths-Jones et al., 2007), we retrieved sequence information for the 962 miRNAs corresponding to their IDs. This sequence information was used as input, and after processing through the Bert model, we obtained a digitized representation of the output, extracting attribute features representing the miRNAs.

### 2.3 Graph embedding for behavioral feature extraction

Graph embedding (Yan et al., 2006) is a technique that maps every node in a diagram structure to a low-dimensional vector space, and it plays a crucial role in many graph data analysis tasks. In the field of bioinformatics, graph embedding is widely used to study complex biological relationships (Yi et al., 2022), such as molecular interactions and gene regulatory networks. Graph embedding utilizes known interactions among circRNAs with miRNAs to obtain a matrix that the behavior feature of circRNAs with miRNAs. An interaction information network is regarded as $G=\left(V,E\right)$ , in which V denotes the collection of vertices (data objects) and E denotes the collection of edges interactions between vertices. Most graph embedding methods only consider first-order proximity, such as Deepwalk (Perozzi et al., 2014). In this study, we employ the Line (Tang et al., 2015) graph embedding algorithm, which preserves both first-order and second-order proximity, thus better preserving the global structure of the network. By learning low-dimensional representations of nodes, we can more effectively capture the similarity and interaction of nodes in the graph structure.

The first-order proximity in the interaction network of circRNA and miRNA is the local pairwise proximity between two vertices, $V_{c}$ and $V_{m}$ . If there is no edge, the first-order proximity is 0. For each undirected edge $\left(c,m\right)$ , the formula for the first-order joint probability distribution of vertices $V_{c}$ and $V_{m}$ is defined as Formula 4:

$$p_{1}\left(V_{c},V_{m}\right)=\frac{1}{1+\exp\left(-{\vec{u}}_{c}^{T}\cdot {\vec{u}}_{m}\right)}$$ (4)

Where ${\vec{u}}_{c},{\vec{u}}_{c}\in R^{d}$ are the low-dimensional representation vectors of vertices $V_{c}$ and $V_{m}$ . It is first-order goal function is shown in Formula 5:

$$O_{1}=\sum_{\left(c,m\right)\in E}W_{cm}\cdot \log p_{1}\left(V_{c},V_{m}\right)$$ (5)

Where $W_{cm}$ is the connection weight between vertices $V_{c}$ and $V_{m}$ . The second-order proximity in the interaction network of CircRNA and miRNA is the similarity between the neighborhood network structures of two vertices $V_{c}$ and $V_{m}$ . If there is no neighborhood network structure, the second-order proximity is 0. In the second-order proximity, each vertex has two tasks: Task 1: the vertex itself, Task 2: a specific context with other vertices. For each edge $\left(c,m\right)$ , the second-order joint probability distribution formula is Formula 6:

$$p_{2}\left(V_{m}|V_{c}\right)=\frac{\exp\left({\vec{u}}_{m}^{\prime T}\cdot {\vec{u}}_{c}\right)}{\sum_{k=1}^{\left|V\right|}\exp\left({\vec{u}}_{k}^{\prime T}\cdot {\vec{u}}_{c}\right)}$$ (6)

where $\left|V\right|$ is the number of vertices or contexts. It is second-order goal function is shown in Formula 7:

$$O_{2}=\sum_{\left(c,m\right)\in E}W_{cm}\cdot \log p_{2}\left(\left(V_{m}|V_{c}\right)\right)$$ (7)

To expedite the learning process, we modify the Formula 7 using negative sampling. The modified objective function is expressed as Formula 8:

$$O_{2}=\log\sigma \left({\vec{u}}_{m}^{\prime T}\cdot {\vec{u}}_{c}\right)+\sum_{i=1}^{K}E_{V_{n}∼P_{n}\left(V\right)}\left[\log\sigma \left(-{\vec{u}}_{n}^{\prime T}\cdot {\vec{u}}_{c}\right)\right]$$ (8)

Where $\sigma \left(x\right)=\frac{1}{\left(1+\exp\left(-x\right)\right)}$ is the sigmoid function, K is the number of negative samples, and $P_{n}\left(V\right)∼d{\left(V\right)}^{\frac{3}{4}}$ is usually set, where $d\left(V\right)$ represents the degree of vertex V .

### 2.4 A neural network model for double layer nested automatic encoder

Autoencoder is an unsupervised learning neural network model designed to learn efficient representations of data. It can be utilized for tasks such as data compression, denoising, and feature extraction. The autoencoder model is instrumental in aiding the exploration and prediction of interactions among non-coding RNAs. In this study, we use the Autoencoder in Autoencoder Networks (Zhang et al., 2019) model to effectively fuse circRNA and miRNA with multi-angle features, that is, to fuse the two angular features of circRNA and miRNA, so that the fused features not only have complementarity between behavioral features and attribute features, but also have complementarity between behavioral features and attribute features. It also has the consistency between behavior characteristics and attribute characteristics. The Autoencoder in Autoencoder Networks model mainly includes the First-AE network and the Second-AE network, which can learn single-angle feature representation and complete multi-angle feature representation together. Then the First AE network is used to extract the implicit information of each Angle automatically, and the degradation process of the Second AE network is used to encode the implicit information of each Angle into the potential representation. We represent the sample of multi-angle features as $X=X^{\left(1\right)},X^{\left(2\right)},...,X^{\left(V\right)}$ , $X^{\left(V\right)}\in R^{d_{v}\times n}$ is the feature matrix of the V−th Angle feature, where V represents the number of angles of the V−th Angle feature, n represents the number of samples of the V−th Angle feature, and $d_{v}$ represents the feature dimension of the V−th Angle feature.

#### 2.4.1 First-AE network

We use $f\left(X^{\left(v\right)};\xi_{ae}^{\left(v\right)}\right)$ to denote the First-AE network for the V−th angle feature, where $\xi_{ae}^{\left(v\right)}={\left\{W_{ae}^{\left(c,v\right)},b_{ae}^{\left(c,v\right)}\right\}}_{c=1}^{C}$ is the parameter set for all layers, $W_{ae}^{\left(c,v\right)}$ represents the relevant weights for the c−th layer, $b_{ae}^{\left(c,v\right)}$ represents the relevant biases for the c−th layer, and C represents the number of layers for nonlinear transformations. The first C/2 encoding layers encode the input feature vector into a new vector, and the last C/2 decoding layers reconstruct the new vector. When the input feature vector is $x_{i}^{\left(v\right)}=z_{i}^{\left(0,v\right)}\in R^{d_{v}}$ , the output of the c−th layer is Formula 9:

$$z_{i}^{\left(c,v\right)}=\sigma \left(W_{ae}^{\left(c,v\right)}z_{i}^{\left(c-1,v\right)}+b_{ae}^{\left(c,v\right)}\right),c=1,2,\ldots ,C$$ (9)

where $\sigma \left(∙\right)$ is the sigmoid activation function, and when the input is the feature matrix $X^{\left(v\right)}=\left[x_{1}^{\left(v\right)},x_{2}^{\left(v\right)},\ldots ,x_{n}^{\left(v\right)}\right]\in R^{d_{v}\times n}$ of the v−th Angle feature, the corresponding reconstruction formula is as Formula 10:

$$Z^{\left(C,v\right)}=\left[z_{1}^{\left(c,v\right)},z_{2}^{\left(c,v\right)},\ldots ,z_{n}^{\left(c,v\right)}\right]$$ (10)

Where $Z^{\left(C,v\right)}$ is the reconstructed representation of the i sample in the v−th Angle feature. We obtain a low-dimensional representation $Z^{\left(\frac{C}{2},v\right)}$ through minimal reconstruction loss, the minimal reconstruction loss is obtained as Formula 11:

$$\min_{{\left\{\xi_{ae}^{\left(v\right)}\right\}}_{v=1}^{V}}\frac{\sum_{v=1}^{V}{\left\|X^{\left(v\right)}-Z^{\left(C,v\right)}\right\|}_{F^{∙}}^{2}}{2}$$ (11)

We encode the obtained low-dimensional angle feature representation $Z^{\left(\frac{C}{2},v\right)}$ into a holistic latent information H for the entire angle feature, where $H\in R^{k\times n}$ and k represents the complete spatial dimension.

#### 2.4.2 Second-AE network

The degradation reduction network of the Second-AE network uses a fully connected neural network (FCNN) to realize that each angular feature can be represented by a new common representation of the whole. We use $g\left(H;\xi_{dr}^{\left(v\right)}\right)$ to represent the degradation restoration network of the v−th angle feature, where $\xi_{ae}^{\left(v\right)}={\left\{W_{dr}^{\left(s,v\right)},b_{dr}^{\left(s,v\right)}\right\}}_{s=1}^{S}$ , and S+1 is the number of layers in the degradation restoration network. We take $H=G^{\left(0,v\right)}$ as input, then $G^{\left(s,v\right)}=\left[g_{1}^{\left(s,v\right)},g_{2}^{\left(s,v\right)},\ldots ,g_{n}^{\left(s,v\right)}\right]$ , where $g_{i}^{\left(s,v\right)}=\sigma \left(W_{dr}^{\left(s\right)}g_{i}^{\left(s-1,v\right)}+b_{dr}^{\left(s,v\right)}\right)$ , and the formula for the goal of degradation reduction is Formula 12:

$$\min_{{\left\{\xi_{dr}^{\left(v\right)}\right\}}_{v=1}^{V}}\frac{\sum_{v=1}^{V}{\left\|Z^{\left(\frac{C}{2},v\right)}-G^{\left(S,v\right)}\right\|}_{F^{∙}}^{2}}{2}$$ (12)

#### 2.4.3 Coupling the First-AE network with the Second-AE network

In the same framework, we learned new vector representations for each angle feature (via the First-AE network) and latent representations for the complete multi-angle features (via the Second-AE network) by coupling the First-AE network with the Second-AE network. The objective function of Autoencoder in Autoencoder Networks model is summarized as Formula 13:

$$\min_{{\left\{\xi_{ae}^{\left(v\right)},\xi_{dr}^{\left(v\right)}\right\}}_{v=1}^{V},H}\frac{\sum_{v=1}^{V}\left[{\left\|X^{\left(v\right)}-Z^{\left(C,v\right)}\right\|}_{F}^{2}+\lambda {\left\|Z^{\left(\frac{C}{2},v\right)}-G^{\left(S,v\right)}\right\|}_{F}^{2}\right]}{2}$$ (13)

Here, λ represents the balance between the consistency and complementarity of multi-angle features.

### 2.5 XGBoost classifier

XGBoost (Chen and Guestrin, 2016) is referred to as extreme gradient boosting, and it is an integrated learning algorithm based on gradient boosting decision trees (GBDT). It is employed to solve machine learning issues such as classification, regression, and ranking. XGBoost benefits from its efficiency, regularization processing, feature importance analysis, and ability to handle missing values, making it a powerful tool for many data science problems. We employ XGBoost as a classifier for predicting circRNA-miRNA interactions. Through multiple iterations, we gradually construct a decision tree model, emphasizing error samples to enhance model performance. The objective function of XGBoost is composed of the loss function and regularization, and is formulated as Formula 14:

$$L^{\left(u\right)}=\sum_{i=1}^{M}l\left(y_{i},{\hat{y}}_{i}^{\left(u-1\right)}+f_{u}\left(x_{i}\right)\right)+\sum_{u}\varphi \left(f_{u}\right)$$ (14)

Here, i represents the i−th sample, u represents the u−th tree, $y_{i}$ is the true value of the i−th sample $x_{i}$ , ${\hat{y}}_{i}$ is the predicted value of the i−th sample $x_{i}$ , l is the differentiable loss function computing the difference between the predicted value ${\hat{y}}_{i}$ and the target value $y_{i}$ , and $\varphi \left(∙\right)$ represents the complexity of the tree. By expanding the second-order Taylor series and regularization term, separately optimizing the loss function term and regularization term, and merging similar terms, the final objective function is obtained as Formula 15:

$$L^{\left(u\right)}=\sum_{j=1}^{U}\left[B_{n}w_{n}+\frac{\left(D_{n}+\lambda \right)w_{n}^{2}}{2}\right]+\gamma U$$ (15)

Here, $B_{n}$ and $D_{n}$ respectively represent the sums of the first and second-order partial derivatives of the samples contained in leaf node n , $w_{n}$ represents the weight of the n−th leaf node, and U represents the number of trees.

## 3 Results

### 3.1 Evaluation indicators criteria

Cross-validation is a commonly used method in the field of machine learning for assessing model performance and reducing the bias of evaluation results. In this work, we employ 5-fold cross-validation (5-fold CV) to assess the predictive power of BJLD-CMI over CMI-9905 dataset. We initially randomly divided the dataset into five subsets, ensuring a balanced distribution of categories in each subset as much as possible. Four subsets were utilized for pieces of training of the model and then one remaining subset was used for validation of the model. This process is repeated five times, ensuring that each subset is used for validation once. The results of the five validations were averaged to get the final performance evaluation metrics (Wang et al., 2018). The experimental evaluation of BJLD-CMI includes accuracy (ACC), precision (Prec.), recall (Rec.), F1-score (F1), and Matthews correlation coefficient (MCC) as reliability assessment criteria. The formula for accuracy is Formula 16, for precision is Formula 17, for recall is Formula 18, for F1-score is Formula 19, and for Matthews correlation coefficient is Formula 20:

$$ACC.=\frac{TN+TP}{TN+TP+FN+FP}$$ (16)

$$Prec.=\frac{TP}{TP+FP}$$ (17)

$$Rec.=\frac{TP}{TP+FN}$$ (18)

$$F1-score=\frac{2\times \left(Precision\times Recall\right)}{Precision+Recall}$$ (19)

$$MCC.=\frac{\left(TP\times TN\right)-\left(FP\times FN\right)}{\sqrt{\left(TP+FP\right)\times \left(TP+FN\right)\times \left(TN+FP\right)\times \left(TN+FN\right)}}$$ (20)

In the above formula, true positives (TP) indicate the sample counts in which the model predicts that circRNAs interact with miRNAs and in which the interaction is realistically confirmed, false positives (FP) indicate the sample counts in which the model predicts that circRNAs interact with miRNAs but in reality are not interaction, true negative (TN) indicates the sample count in which the model predicts that circRNAs not interact with miRNAs and realistically confirms that there is no interaction, false negative (FN) indicates the sample count in which the model predicts that circRNAs not interact with miRNAs but the interaction is confirmed in reality. We also plotted the Receiver Operating Characteristic (ROC) curve of BJLD-CMI under 5-fold cross-validation (Zweig and Campbell, 1993) and computed the AUC to evaluate the performance of the models.

### 3.2 Evaluation model prediction ability

**FIGURE 3:**
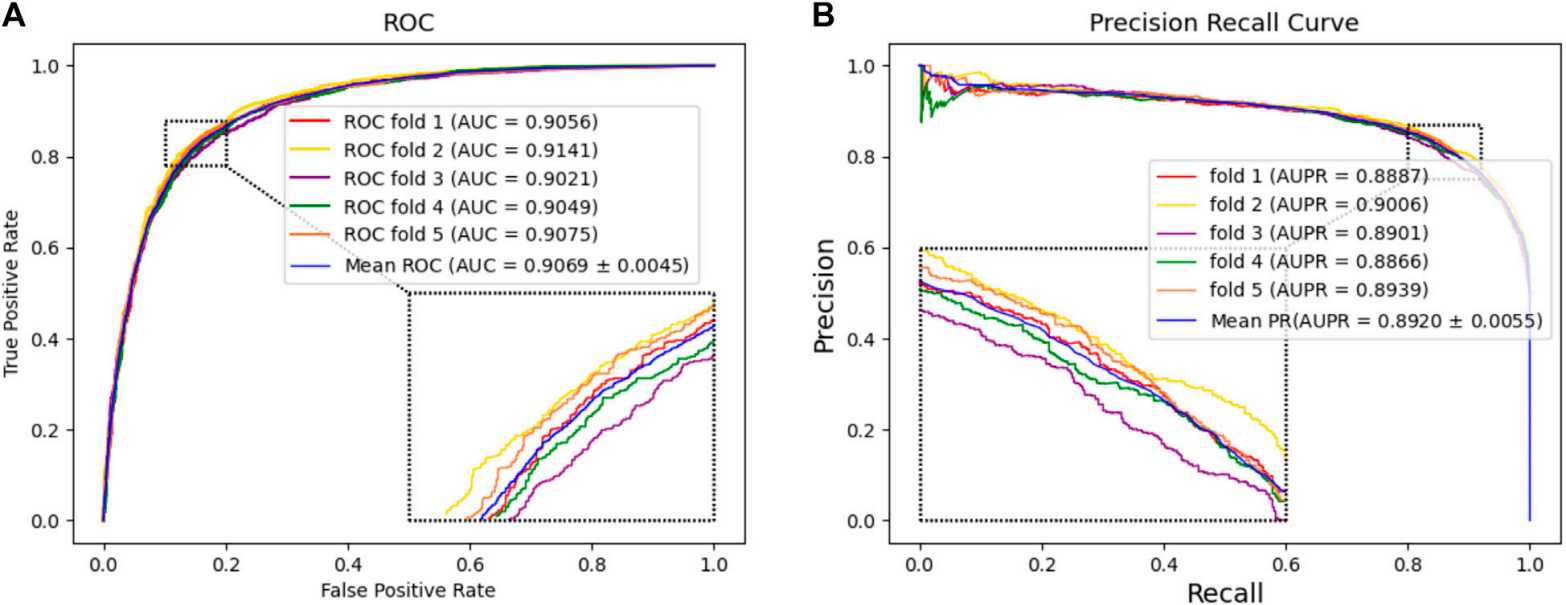
Performance evaluation of BJLD-CMI **(A)** ROC curve obtained by BJLD-CMI on the CMI-9905 dataset **(B)** PR curve obtained by BJLD-CMI on the CMI-9905 dataset.

In this experiment, we tested the performance of BJLD-CMI in predicting circRNA-miRNA interactions over the CMI-9905 dataset with a 5-fold cross-validation method. Table 2 lists the details of the experimental results. From Table 2, we can see that our model obtained a mean accuracy of 83.41%. The accuracies for the five experiments were 83.47%, 84.07%, 82.53%, 83.06%, and 83.90% respectively, with a standard deviation of 0.56%. In the evaluation criteria of precision (Prec), recall (Rec), F1-score, and Matthews correlation coefficient (MCC), BJLD-CMI demonstrated an accuracy of 85.31%, 80.70%, 82.94%, and 66.91%, with respective standard deviations of 0.38%, 0.93%, 0.64%, and 1.10%. In addition, we also computed the AUC and AUPR that BJLD-CMI generated over the CMI-9905 dataset with their ROC curves and PR curves plotted. Concerning AUC, the five experiments yielded results of 90.56%, 91.41%, 90.21%, 90.49%, and 90.75%, with a mean value of 90.69% and a standard deviation of 0.45%. Concerning AUPR, the five experiments resulted in 88.87%, 90.06%, 89.01%, 88.66%, and 89.39% with a mean value of 89.20% and a standard deviation of 0.55%, respectively. Figure 3A shows the ROC curves generated by five experiments, and Figure 3B shows the PR curves generated by five experiments. Through the experimental results described above, it is clearly observed that the BJLD-CMI is able to predict CMI effectively on CMI-9905, and shows excellent comprehensive performance, exhibits good application prospects, and may be considered as a potential tool for exploring the unknown CMI.

**TABLE 2:** Cross-validation results of BJLD-CMI on the CMI-9905 dataset.

Test set	ACC. (%)	Prec. (%)	Rec. (%)	F1-score (%)	MCC. (%)	AUC (%)
1	83.47	85.12	81.12	83.07	67.01	90.56
2	84.07	85.94	81.47	83.65	68.24	91.41
3	82.53	84.93	79.10	81.91	65.22	90.21
4	83.06	85.03	80.26	82.58	66.23	90.49
5	83.90	85.55	81.57	83.51	67.87	90.75
Average	83.41 ± 0.56	85.31 ± 0.38	80.70 ± 0.93	82.94 ± 0.64	66.91 ± 1.10	90.69 ± 0.45

### 3.3 Evaluation comparison of different dimensions of line

**FIGURE 4:**
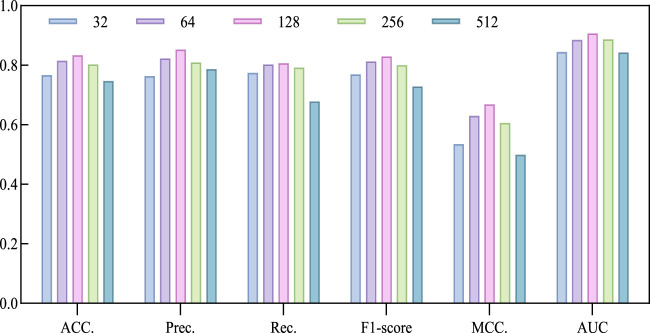
Performance bar visualization comparison with different Line dimensions on CMI-9905 dataset.

The model utilizes the Line algorithm in graph embedding to learn low-dimensional embeddings of nodes in the circRNA-miRNA relationship network, preserving the network structure and relationships between nodes. Determining the dimension is a crucial factor in describing the features of circRNA and miRNA. Choosing a big dimension could increase the computational workload and intricacy of the suggested model, resulting in longer execution times and potentially lower accuracy. Conversely, selecting a small dimension might lead to an insufficient feature extraction. Therefore, we chose a series of commonly used dimensions, specifically 32, 64, 128, 256, 512, and conducted a 5-fold cross-validation experiment to select the optimal dimension. Based on the experimental results in Table 3; Figure 4, we observed a continuous improvement in the overall performance of the model with increasing dimensions. When the dimension parameter increases to 128, the model achieves optimal performance, as reflected in the maximum values of ACC, AUC, Prec., and MCC. However, when the dimension exceeds 128, the performance gradually declines. Therefore, we decided to fix the dimension parameter at 128.

**TABLE 3:** Results of 5-fold cross-validation on CMI-9905 dataset with different Line dimensions.

Dimensions	Mean ACC	Mean Prec	Mean Rec	Mean F1-score	Mean MCC	Mean AUC
32	0.7675	0.7637	0.7748	0.7692	0.5351	0.8449
64	0.8151	0.8232	0.8026	0.8127	0.6303	0.8858
128	0.8341	0.8531	0.8070	0.8294	0.6691	0.9069
256	0.8029	0.8094	0.7923	0.8008	0.6059	0.8869
512	0.7473	0.7867	0.6786	0.7287	0.4994	0.8431

### 3.4 Comparison of different classifiers

**FIGURE 5:**
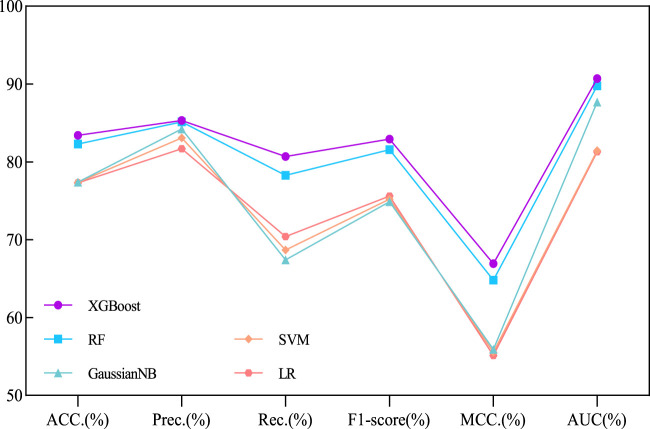
Performance comparison line chart of different traditional classifiers and XGBoost on the CMI-9905 dataset.

Our proposed BJLD-CMI model uses the XGBoost classifier for data training and classification tasks over the CMI-9905. To validate the performance of the XGBoost classifier, we replaced it with four other classifiers while keeping the dataset and other conditions unchanged. These classifiers are Random Forest (RF) (Breiman, 2001), Gaussian Naive Bayes (GaussianNB), Support Vector Machine (SVM) (Suykens and Vandewalle, 1999), and Logistic Regression (LR) (Dreiseitl and Ohno-Machado, 2002). The performance of these five classifiers in predicting CMI was evaluated by 5-fold cross-validation and their categorization performance is compared. Table 4 concludes the average results of the five classifiers combined with the CMI-9905 dataset after 5-fold cross-validation and is presented in line graph form. From Figure 5, we can intuitively observe that XGBoost achieved the highest results in six evaluation metrics, including ACC, Prec, Rec, F1, MCC, and AUC. This indicates that XGBoost performs better in predicting unknown CMI in the proposed model.

**TABLE 4:** Results of different traditional classifiers and XGBoost in 5-fold cross-validation on CMI-9905 dataset.

Classifier	Testing set	ACC. (%)	Prec. (%)	Rec. (%)	F1-score (%)	MCC. (%)	AUC (%)
XGBoost	Average	83.41	85.31	80.70	82.94	66.91	90.69
	SD	0.56	0.38	0.93	0.64	1.10	0.45
RF	Average	82.30	85.14	78.26	81.56	64.82	89.77
	SD	0.30	0.35	0.75	0.39	0.58	0.36
GaussianNB	Average	77.40	84.24	67.40	74.88	55.92	87.68
	SD	0.78	0.65	1.51	1.05	1.46	0.54
SVM	Average	77.36	83.09	68.69	75.21	55.55	81.44
	SD	0.64	0.88	1.02	0.76	1.28	0.80
LR	Average	77.30	81.67	70.41	75.62	55.13	81.30
	SD	0.19	0.30	0.33	0.22	0.39	0.46

### 3.5 Comparison of the existing method

Currently, numerous outstanding computational approaches have been proposed, relying on benchmark datasets CMI-9589 [from the Circbank database (Liu et al., 2019)] and CMI-9905 [from the Circbank database (Liu et al., 2019) and Circr2cancer database (Lan et al., 2020)]. These methods include WSCD (Guo et al., 2022), SGCNCMI (Yu et al., 2022), KGDCMI (Wang et al., 2022b), DeepCMI (Li et al., 2023), CMIVGSD (Qian et al., 2021b), GCNCMI (He et al., 2022), JSNDCMI (Wang et al., 2023), aim to forecast potential CMIs. In order to further assess BJLD-CMI’s predictive performance, we have compared it to these methods in two datasets, respectively. For fairness, we chose the AUC generated by the fivefold CV method as the parameter for evaluation. Table 5 presents the contrasting outcomes of CMIVGSD, SGCNCMI, KGDCMI, GCNCM, JSNDCMI, and DeepCMI with BJLD-CMI utilizing the CMI-9589 dataset. Table 6 shows the contrasting outcomes of KGDCMI, WSCD, SGCNCM, JSNDCMI, and DeepCMI with BCMCMI utilizing the CMI-9905 dataset. The results in Tables 5, 6 indicate that our model achieved the highest AUC results, surpassing the averages by 0.00986 and 0.03158, respectively. Overall, BJLD-CMI demonstrates strong competitiveness among existing methods.

**TABLE 5:** AUC values of related models and BJLD-CMI on 5-fold cross-validation in CMI-9589 dataset.

Models	CMIVGSD	SGCNCMI	KGDCMI	GCNCMI	JSNDCMI	DeepCMI	BJLD-CMI
AUC	0.8804	0.9015	0.9041	0.9320	0.9415	0.9480	0.9495
AUPR	0.8629	0.9011	0.8937	0.9396	0.9403	0.9416	0.9474

**TABLE 6:** AUC values of related models and BJLD-CMI on 5-fold cross-validation in CMI-9905 dataset.

Models	KGDCMI	WSCD	SGCNCMI	JSNDCMI	DeepCMI	BJLD-CMI
AUC	0.8930	0.8923	0.8942	0.9003	0.9054	0.9069
AUPR	0.8767	0.8935	0.8887	0.8999	0.8978	0.8920

### 3.6 Case studies

In order to validate the genuine predictive ability of BJLD-CMI for miRNA-associated circRNA, we performed a case study utilizing the CMI-9905 dataset. In the experiment, we trained the BJLD-CMI model using known CMIs extracted from the CMI-9905 dataset and then used the trained model to predict unknown CMIs. Following the acquisition of the prediction outcomes, we organized the prediction scores in descending order and validated the top 10 circRNA-miRNA pairs in the published literature. The specific findings are outlined in Table 7. From the table, we can conclude that 7 pairs have been confirmed in PubMed, confirming the involvement of circRNA as miRNA sponges in the biological processes of diseases such as lung cancer (Wang et al., 2019; Yao et al., 2019; Zhou et al., 2019), prostate cancer (Wu et al., 2019), and gastric cancer (Zhong et al., 2018; Liang et al., 2019). It’s worth noting that the lack of confirmation in existing literature for the other 4 pairs does not necessarily negate the possibility of an interaction between them. The results of the case study indicate that BJLD-CMI is a powerful tool with the prospect of exploring the interaction of unknown circRNAs with miRNAs.

**TABLE 7:** Top 10 CMI pairs predicted by BJLD-CMI.

Num	miRNA	circRNA	Evidence	Cancer
1	hsa-miR-1183	hsa_circ_0004015	PMID:30509491	Non-Small Cell Lung Cancer
2	hsa-miR-4667-3p	hsa_circ_0002172	Unconfirmed	Unconfirmed
3	hsa-miR-135a-5p	hsa_circ_0001946	PMID:30841451	Lung Adenocarcinoma
4	hsa-miR-181c-5p	hsa_circ_0001427	PMID:30674872	Prostate Cancer
5	hsa-miR-139-3p	hsa_circ_0000592	PMID:31189743	Gastric Cancer
6	hsa-miR-638	hsa_circ_0000177	PMID:30010402	Glioma
7	hsa-miR-1224-3p	hsa_circ_0001731	Unconfirmed	Unconfirmed
8	hsa-miR-214-5p	hsa_circ_0000993	PMID:30215537	Gastric Cancer
9	hsa-miR-619-5p	hsa_circ_0004939	Unconfirmed	Unconfirmed
10	hsa-miR-330-5p	hsa_circ_0001727	PMID:32010565	Non-Small Cell Lung Cancer

## 4 Conclusion

With the popularity of computational models and the booming development of bioinformatics, people are gradually realizing the importance of the associative relationships between circRNAs and miRNAs in various biological processes as well as in the treatment of diseases. By applying computational models to predict CMI, we can get a deeper understanding of the unrevealed hidden networks between circRNAs and miRNAs, and thus study their roles in regulating gene expression and participating in organic processes. Exploring the correlation between circRNAs and miRNAs provides biologists with new ideas, which are important clinical guidance for the diagnosis and treatment of diseases.

On this work, we suggest a computationally grounded model, BJLD-CMI, to forecast circRNA with miRNA interaction relationships. In this model, we first convert miRNA sequences into digital representations using natural language processing techniques, apply Jaccard similarity coefficients to obtain the feature expressions of circRNAs through the moving window method, and construct the corresponding attribute feature matrices from the known circRNA with miRNA relationship pairs. Subsequently, from the known circRNA with miRNA relationship network, we build the corresponding behavioral feature matrix using the graph embedding method Line. In addition, the Autoencoder in Autoencoder Networks model is used to learn the new vector representation of each Angle feature and the potential representation of the complete multi-angle feature respectively from the perspective of the behavior and attribute features of circRNA and miRNA, so that the obtained features not only have the complementarity between the behavior feature and the attribute feature but also have the consistency. On the CMI-9589 and CMI-9905 datasets, BJLD-CMI achieved excellent results using the XGBoost classifier. To evaluate the performance of BJLD-CMI, we conducted experiments comparing different classifiers and experiments comparing with other models. The results indicate that BJLD-CMI outperforms other models. We also conducted a case study, and among the top ten ranked circRNA-miRNA pairs in prediction scores, 7 pairs were verified in our literature search on PubMed. This provides new insights for research on diseases such as non-small cell lung cancer, lung adenocarcinoma, prostate cancer, and gastric cancer.

These results indicate that the BJLD-CMI model can predict the underlying relationship between circRNAs and miRNAs efficiently and is a reliable predictive model, but there are still some limitations. Firstly, the BJLD-CMI model is dependent on the amount of data on known circRNA-miRNA interaction, and too large a gap of positive and negative samples can have a significant impact on the correctness of the model’s predictions. Secondly, different feature extraction methods and parameter settings may also impact the model’s predictions. Additionally, the BJLD-CMI model could not make direct predictions for circRNA-miRNA pairs that have no known interactions. In future research, we will continue exploring the application of NLP in extracting information from biological sequence data and integrating additional perspectives of biological feature information to enhance the accuracy and reliability of the model.

## Data Availability

The datasets used in this paper can be found in the CircR2Cancer database (http://www.biobdlab.cn:8000/) and the CircBank database (http://www.circbank.cn/). The source code can be found at https://github.com/YXzhaok/BJLDCMI.
